# Advanced glycation end products are elevated in estrogen receptor-positive breast cancer patients, alter response to therapy, and can be targeted by lifestyle intervention

**DOI:** 10.1007/s10549-018-4992-7

**Published:** 2018-10-27

**Authors:** Katherine R. Walter, Marvella E. Ford, Mathew J. Gregoski, Rita M. Kramer, Kendrea D. Knight, Laura Spruill, Lourdes M. Nogueira, Bradley A. Krisanits, Van Phan, Amanda C. La Rue, Michael B. Lilly, Stefan Ambs, King Chan, Tonya F. Turner, Heidi Varner, Shweta Singh, Jaime Uribarri, Elizabeth Garrett-Mayer, Kent E. Armeson, Ebony J. Hilton, Mark J. Clair, Marian H. Taylor, Andrea M. Abbott, Victoria J. Findlay, Lindsay L. Peterson, Gayenell Magwood, David P. Turner

**Affiliations:** 1 Department of Pathology & Laboratory Medicine, Medical University of South Carolina (MUSC), Charleston, SC USA; 2 Department of Public Health Sciences, MUSC, Charleston, SC USA; 3 Hollings Cancer Center, MUSC, Charleston, SC USA; 4 Department of Exercise Science, College of Arts and Sciences, Campbell University, Buies Creek, NC USA; 5 Ralph H. Johnson Veterans Affairs Medical Center, Charleston, SC USA; 6 Laboratory of Human Carcinogenesis, Center for Cancer Research, National Cancer Institute, Bethesda, MD USA; 7 Cancer Research Technology Program, Leidos Biomedical Research, Frederick National Laboratory, Frederick, MD USA; 8 Dietetic Services, MUSC, Charleston, SC USA; 9 Department of Medicine/Renal Medicine, Icahn School of Medicine at Mount Sinai, New York, NY USA; 10 Department of Anesthesia and Perioperative Medicine, MUSC, Charleston, SC USA; 11 Department of Medicine, Division of Cardiology, MUSC, Charleston, SC USA; 12 Department of Surgery, MUSC, Charleston, SC USA; 13 Washington University School of Medicine, St. Louis, MO USA; 14 College of Nursing, MUSC, Charleston, SC USA; 15 James E. Clyburn Research Center Medical University of South Carolina, Charleston, SC 29425 USA

**Keywords:** Advanced glycation end product, Estrogen receptor, Breast cancer, Tamoxifen resistance, Lifestyle intervention

## Abstract

**Purpose:**

Lifestyle factors associated with personal behavior can alter tumor-associated biological pathways and thereby increase cancer risk, growth, and disease recurrence. Advanced glycation end products (AGEs) are reactive metabolites produced endogenously as a by-product of normal metabolism. A Western lifestyle also promotes AGE accumulation in the body which is associated with disease phenotypes through modification of the genome, protein crosslinking/dysfunction, and aberrant cell signaling. Given the links between lifestyle, AGEs, and disease, we examined the association between dietary-AGEs and breast cancer.

**Methods:**

We evaluated AGE levels in bio-specimens from estrogen receptor-positive (ER+) and estrogen receptor-negative (ER−) breast cancer patients, examined their role in therapy resistance, and assessed the ability of lifestyle intervention to reduce circulating AGE levels in ER+ breast cancer survivors.

**Results:**

An association between ER status and AGE levels was observed in tumor and serum samples. AGE treatment of ER+ breast cancer cells altered ERα phosphorylation and promoted resistance to tamoxifen therapy. In a proof of concept study, physical activity and dietary intervention was shown to be viable options for reducing circulating AGE levels in breast cancer survivors.

**Conclusions:**

There is a potential prognostic and therapeutic role for lifestyle derived AGEs in breast cancer. Given the potential benefits of lifestyle intervention on incidence and mortality, opportunities exist for the development of community health and nutritional programs aimed at reducing AGE exposure in order to improve breast cancer prevention and treatment outcomes.

**Electronic supplementary material:**

The online version of this article (10.1007/s10549-018-4992-7) contains supplementary material, which is available to authorized users.

## Introduction

Breast cancer is the 2nd leading cause of new cancer cases in the US, where more than 3 million breast cancer survivors reside. Lifestyle behaviors such as an unhealthy diet and physical inactivity are modifiable prognostic factors that may have distinct molecular consequences on breast cancer outcomes. Despite a current consensus that exercise and a healthy diet may improve breast cancer prognosis, little is known about their positive effects on the biological pathways involved in breast tumor biology.

Advanced glycation end products (AGEs) are reactive metabolites produced endogenously as a by-product of sugar metabolism as well as the oxidation of biological molecules [1–3]. AGEs irreversibly accumulate in our tissues causing pathogenic effects on organ homeostasis, genetic fidelity, protein function, and cell signaling cascades [1–3]. Significantly, an unhealthy diet, high in sugar, fat, and highly processed foods along with a sedentary lifestyle also contribute to the AGE accumulation pool to contribute to chronic disease development and complications [4–6]. Increases in the AGE accumulation pool lead to the perpetual activation of cancer-associated cell signaling cascades including MAPK (mitogen-activated protein kinase) and AKT (protein kinase B) leading to aberrant transcriptional activity, immune function, and oxidative stresses [7].

The pathogenic effects of AGEs are often mediated through activation of the cognate receptor for AGE (RAGE) [1–3, 6]. RAGE knockdown in ER+ MCF7 cells decreases 17α-ethinyl-estradiol-dependent proliferation and survival through changes in AKT, CCND1 (cyclin D1), and BCL2 (B-cell lymphoma 2) [8]. AGE-RAGE signaling promotes the activation of redox-responsive transcription factors including NFkB (nuclear factor kappa-light-chain-enhancer of activated B cells) and AP-1 (activator protein 1) to increase MAPK and ERK (extracellular signal-regulated kinase) signaling [9]. Crosstalk between ERα and NFkB can stimulate cell proliferation and survival and contribute to tamoxifen resistance [10]. Recent data also indicate that inhibition of ERα by endocrine therapies such as tamoxifen may release NFκB from ERα-driven inhibition, possibly resulting in tamoxifen resistance and NFκB-driven tumor progression [11]. Additionally, several outcome signatures derived from women treated with tamoxifen have implicated immune response as a common mechanism contributing to tamoxifen resistance [12–14].

Given the common signaling cascades involved in AGE pathogenesis and ERα regulation, we examined the ability of AGEs to augment ERα phosphorylation and tamoxifen resistance, and tested the ability of a defined lifestyle intervention program to lower AGE levels in overweight/obese post-menopausal women with non-metastatic stage I–III ER+ breast cancer.

## Methods

### Retrospective biological samples

For AGE analysis, serum samples were obtained from the Hollings Cancer Center Tissue Biorepository at the Medical University of South Carolina (MUSC). In total, 20 well and 20 poorly differentiated serum samples (1 ml of each) were selected from female breast cancer patients which had been stored between 1 and 5 years since diagnosis.

Tissue microarray’s were obtained from US BioMax (Rockville, MD) and contained 96 samples of non-cancer, hyperplastic, and cancerous tissue from 48 patients. Pathological data available from each patient included tumor grade and receptor status. Tissue for mass spectrometry analysis was obtained from breast cancer patients recruited in Baltimore hospitals between 1993 and 2003, as previously described [15, 16].

### Immunohistochemical (IHC) staining

IHC staining was performed to examine AGE levels in tissue as previously published [4]. IHC scores were calculated using the formula: intensity X % positive tumor cells. Intensity staining was scored as follows: 1—weak, 2—intermediate, 3—strong, and 4—very strong. Percent positive tumor cells were scored as follows: 1—less than 10%, 2—10–30%, 3—30–60%, and 4—60–100% positive cells by a pathologist.

### AGE ELISA

To examine the levels of AGEs in serum, 96-well format Oxi-select ELISA’s (Cell Biolabs, San Diego, CA) were used as directed by the manufacturer. All samples were normalized to total protein concentration [4].

### Mass spectrometry analysis

Carboxymethyllysine (CML) and carboxyethyllysine (CEL) and the internal standards (IS) CML-^13^C_4_/CEL-^13^C_4_ were obtained from Sigma-Aldrich/Santa Cruz. The CML/CEL stock solutions were prepared in water as 1 mg/mL solutions. The CML/CEL calibration standards, 0.8–100 ng/mL, were prepared by serial dilution of the stocks with PBS. The working IS solution (10 ng/mL each of the ^13^C_4_) was prepared by diluting the IS stocks with 0.24N trichloroacetic acid (TCA) solution. The tissue samples were wet weighted and homogenized in PBS using the Bead Ruptor (Omni), followed by centrifugation (10 min/16 kg). 15 µL of the tissue supernatant or calibration standards were vortex mixed with 75 µL IS solution. After centrifugation (10 min/16 kg), the solutions were transferred to injection vials for LC/MS/MS. LC was performed with a Shimadzu 20AC-XR system. The injection volume was 40 µL. Separation was achieved with a 2 × 150 mm, 3 µm SS-C18 column (Imtakt). Mobile phase A was 0.1% formic acid in water and mobile phase B was 50 mM ammonium acetate/80% methanol. The flow rate was 300 µL/min and peaks were eluted with a 8-min gradient. MS/MS was performed with a TSQ Vantage triple quadrupole mass spectrometer (Thermo Fisher Scientific) operating in selected reaction monitoring (SRM) mode with positive electrospray ionization. The target peaks were detected using the following m/z precursor > product ions: CML (205 > 84); CML-^13^C_4_ (209 > 88); CEL (219 > 84); CEL-^13^C_4_ (223 > 88). CML and CEL in the supernatant (Y ng/mL) were determined using the Thermo Xcalibur software. Calibration curves, constructed by plotting the peak area ratios vs. standards, were fitted by linear regressions (*R*^2^ = 0.99). The peak area ratios were calculated by dividing the peak areas of CML or CEL by the peak area of CML-^13^C_4_ or CEL-^13^C_4_, respectively.

### Cell culture

MCF7 and T47D cell lines were purchased from ATCC (Manassas, VA). Cells were incubated at 37 °C, 5% CO_2_ in their respective media. Cells were cultured up to 30 passages before being replaced from low passage stocks. Mycoplasma-negative cultures were ensured by PCR testing. Cells were monitored throughout with consistent morphology and doubling-time. T47D cells were incubated in RPMI (Fisher Scientific, Fair Lawn, NJ) with 10% fetal bovine serum (Fisher Scientific) and 1% Penicillin/Streptomycin (Fisher Scientific). MCF7 cells were incubated in DMEM/High Glucose media (Fisher Scientific) containing 10% FBS and 1% Penicillin/Streptomycin with the following additives: 1% MEM Non-essential Amino Acids, 1% Sodium Pyruvate, 1% Sodium Bicarbonate, and 1% insulin (all obtained from Life Technologies, Grand Island, NY).

### Exogenous AGE treatment

Exogenous BSA-AGE and control BSA were produced as previously published [17]. For examination of AGE treatment, cells (400,000) were seeded into each well of a 6-well plate in serum-free media overnight before treatment with AGE metabolite (50ug/ml). Effects of AGE treatment on the phosphorylation of ERα, AKT, and ERK was assessed by Western blot as indicated below. Pharmacological targeting of the MAPK/ERK and PI3K/AKT pathways in the presence of AGE was achieved in serum-free media using the molecular inhibitors U0126 (ERK) and LY294002 (AKT) (Cell signaling Technology, Danvers, MA) at a concentration of 10uM for 12 h before exposure to AGE metabolites. To examine the effects of AGE metabolite on tamoxifen resistance, cells (3,000) were seeded into each well of a 96-well plate and were treated with the active metabolite of tamoxifen, 4-hydroxytamoxifen (0, 10 µM) (Sigma-Aldrich, St. Louis, MO) in combination with varying doses of AGEs (0, 5 µg/mL, 10 µg/mL, 50 µg/mL). After 24, 48, and 72 h, SRB staining was used to quantify cell growth as described previously [18].

### Western Blot analysis

For isolation of total protein, cells at 70–80% confluence were washed twice with ice cold 1xPBS, and were lysed in radio-immunoprecipitation assay (RIPA) buffer containing protease and phosphatase inhibitors (Sigma). Equal amounts of total protein (50 µg) were resolved by 10% SDS-PAGE and subjected to Western blot analyses using ECL system (Pierce-Fisher Scientific, Rockford, IL). Total protein lysates were examined using primary antibodies against total ERα, phosphorylated ERα-ser118 (p-ERα (ser118)), phosphorylated ERα-ser167 (p-ERα (ser167)), total ERK, phosphorylated-ERK (pERK), total AKT, phosphorylated-AKT (pAKT), and GAPDH (all obtained from Cell Signaling Technology).

### Intervention design and dietary assessment

See supplementary methods.

### Statistical analysis

See supplementary methods.

## Results

### AGE levels are elevated in ER+ breast cancer patients

**Fig. 1:**
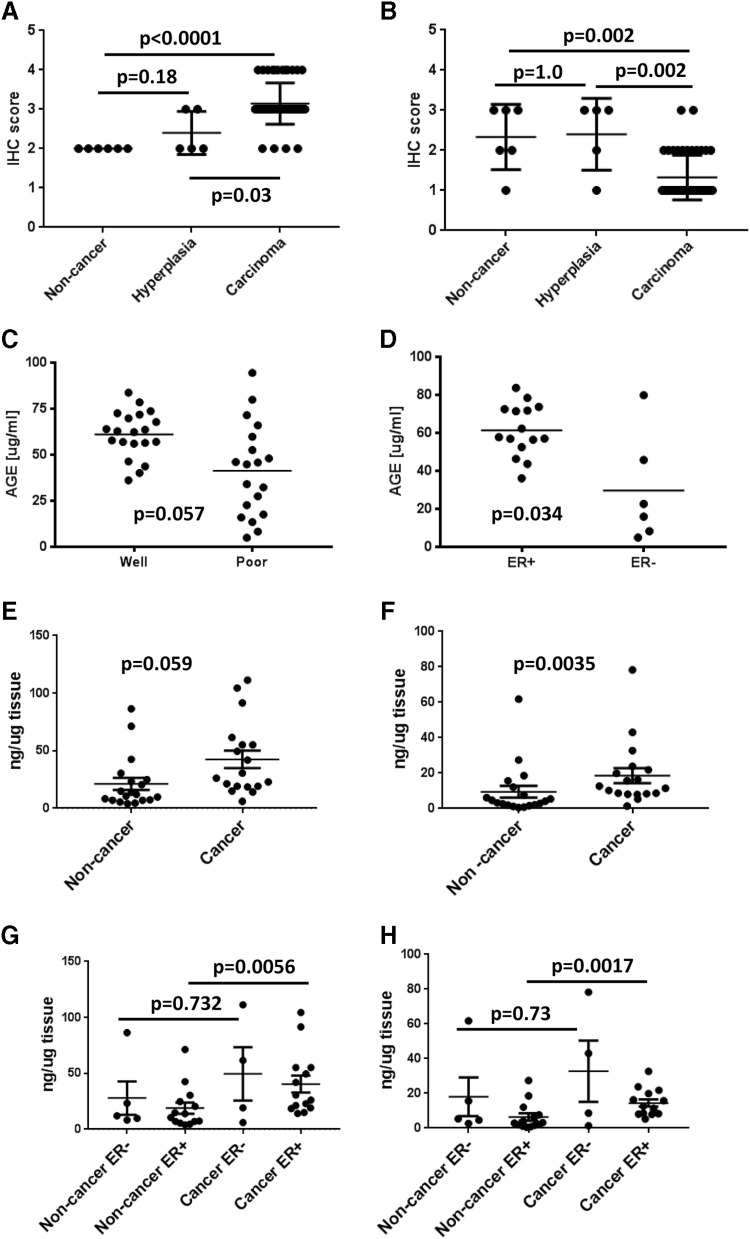
AGE levels are elevated in ER+ breast cancer patients. **a** Quantification of epithelial AGE levels in breast normal and tumor tissue using IHC staining. **b** Quantification of stromal AGE levels in breast tumor tissue using IHC staining. **c** Quantification of circulating AGE levels, analyzed by ELISA, in serum from breast cancer patients stratified by well and poorly differentiated tumors. **d** Quantification of circulating AGE levels, analyzed by ELISA in serum from breast cancer patients stratified by ER status. **e** Quantification of CML levels in non-cancer and breast tumor tissue using LC/MS/MS. **f** Quantification of CEL levels in non-cancer and breast tumor tissue using LC/MS/MS. **g** Quantification of CML levels, analyzed by LC/MS/MS in non-cancer and breast tumor tissue stratified by ER status. **h** Quantification of CEL levels, analyzed by LC/MS/MS in non-cancer and breast tumor tissue stratified by ER status

Breast tumor microarrays were stained using antibodies specific for AGE. Staining intensity in tumor tissue varied by stage and differentiation (Fig. 1). Highest staining for epithelial AGE was observed in carcinoma and then hyperplasia tissue when compared to non-cancer tissue specimens (Fig. 1a). In contrast, stromal AGE in the same tissue samples was highest in non-cancer and hyperplasia tissue (Fig. 1b). In a separate set of serum samples obtained from the MUSC biorepository, analysis by ELISA shows that circulating AGE levels are higher in serum from patients with well-differentiated tumors compared to poorly differentiated (Fig. 1c). When stratified by ER status, ER+ patients had significantly higher circulating AGE levels than ER- patients (Fig. 1d).

### N^ε^-carboxymethyllysine and N^ε^-carboxyethyllysine are elevated in breast tumors

Levels of CML and CEL were assessed in a separate set of 19 non-cancerous and 18 cancerous breast tissue samples using liquid chromatography–tandem mass spectrometry (LC/MS/MS). Compared to non-cancerous tissue, both CML (Fig. 1e) and CEL (Fig. 1f) levels were elevated in the tumor tissues. Average levels of CML and CEL in the tumor samples were 42.44 ng/µg and 18.55 ng/µg, respectively, compared to 21.29 ng/µg and 9.468 ng/µg in non-cancerous tissue. When stratified by ER status both CML and CEL levels were higher in ER+ and ER− tumor tissue compared to non-cancerous but this only reached statistical significance for the ER+ comparison (Fig. 1g, h). Collectively these data show that both CML and CEL are elevated in the tumor of breast cancer patients and may trend towards higher levels in ER+ patients.

### AGE treatment activates pathways associated with ER regulation and tamoxifen resistance

**Fig. 2:**
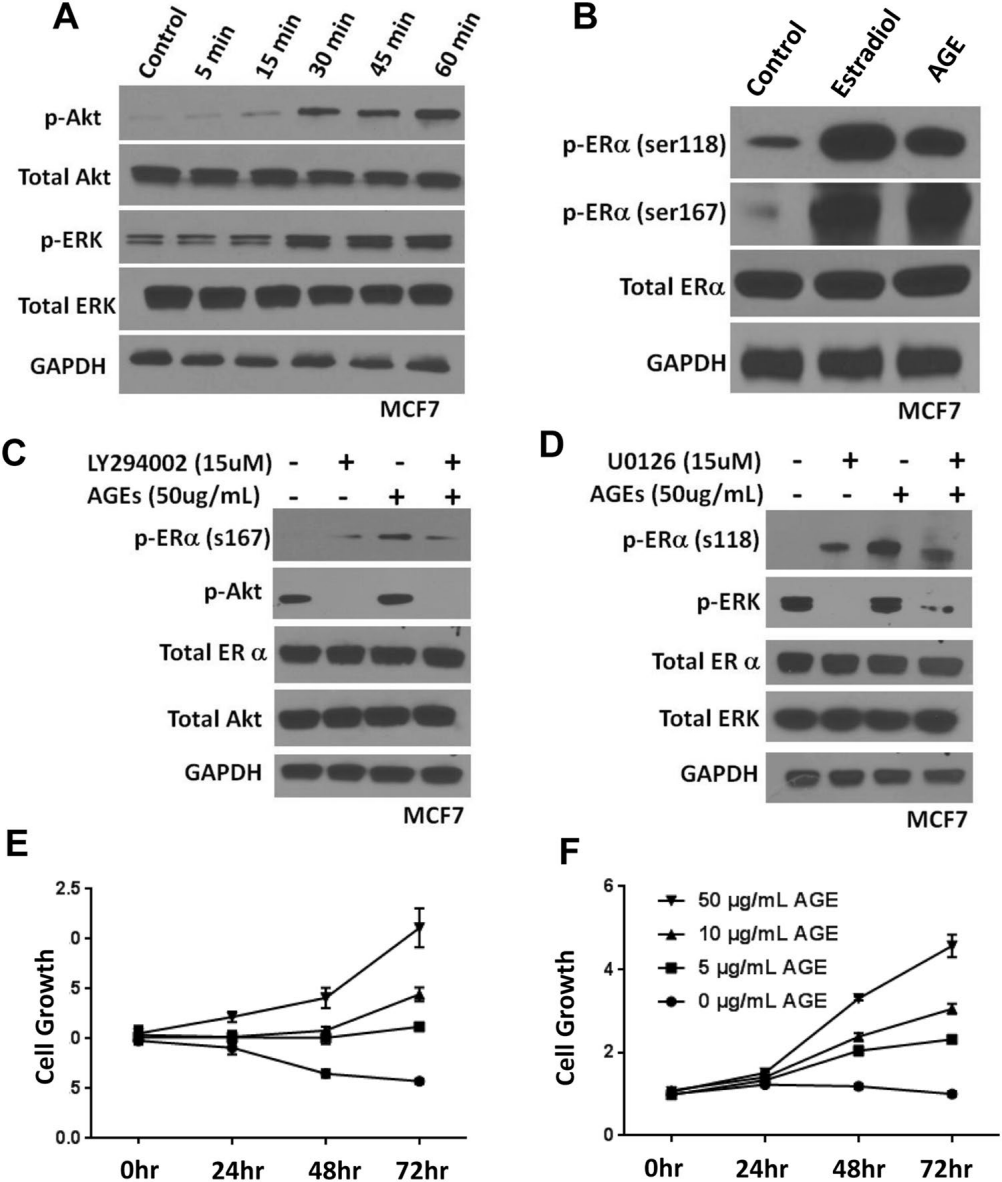
AGE treatment activates pathways associated with ER regulation and tamoxifen treatment. **a** Western blot analysis of time-dependent AKT and ERK phosphorylation after treatment with AGE (50ug/mL) in MCF7 breast cancer cells. **b** Western blot analysis of ERα phosphorylation after treatment with AGE (50ug/mL) in MCF7 breast cancer cells. **c** Western blot analysis of AKT and ERK phosphorylation after treatment with AGE (50ug/mL) in MCF7 cells in the presence of AKT inhibitor. **d** Western blot analysis of AKT and ERK phosphorylation after treatment with AGE (50ug/mL) in MCF7 cells in the presence of ERK inhibitor. **e** Cytotoxicity assay of MCF7 cell proliferation after treatment with tamoxifen (10uM) in the presence of increasing AGE concentration. **f** Cytotoxicity assay of MCF7 cell proliferation after treatment with tamoxifen (20 μM) in the presence of increasing AGE concentration

We first examined whether AGE stimulates AKT and ERK activity in ER+ breast cancer cells. Cells were treated with BSA-AGE (50 μg/mL), and the effects on AKT (ser473) and ERK phosphorylation (ser202/tyr204) assessed. Control cells were treated with BSA [17]. We observed a significant increase in both AKT and ERK phosphorylation in both MCF7 (Fig. 2a) and T47D (Supplementary Fig. 1a) cell lines in response to AGE treatment in a time-dependent manner.

Phosphorylations of ser118 and ser167 within the ligand-independent activation domain of ERα are two residues emerging as potential predictive markers for patient response to tamoxifen [19, 20]. MCF7 and T47D cells were treated with 50ug/mL of AGEs for 30 min and were then examined for phosphorylation of ERα at residues ser118 and ser167. 100 nM estradiol, one of the key estrogenic ligands for the receptor, was used as a positive control to ensure optimization of phospho-specific antibodies. In both cell lines and at both residues, we found a significant increase in phosphorylation of ERα following AGE treatment when compared to untreated control with no overall change in total ERα levels (Fig. 1b and Supplementary Fig. 1b). We also examined the influence of AGE treatment on the phosphorylation of ERα at ser167 over a time course of 1 h in MCF7 and T47D cells and observed that the AGE-mediated phosphorylation of ERα is transient in nature with a peak around 15–30 min following initial treatment (Supplementary Fig. 1c, d).

To determine if ERK and AKT signaling are required for AGE-mediated ERα phosphorylation, both ERK and AKT pathways were pharmacologically inhibited in the presence of AGE. In MCF7 cells, inhibition of AKT with Ly294002 and inhibition of ERK with the MEK inhibitor U0126 significantly reduced phosphorylation of ERα ser167 (Fig. 2c) and ser118 (Fig. 2d), respectively, in the presence (50ug/mL) of AGE metabolite. Similar results were observed in T47D cells treated in the same manner (Supplementary Fig. 1e, f).

To explore a potential role for AGE metabolites in tamoxifen resistance, we treated MCF7 and T47D cells with varying levels of AGEs in the presence of tamoxifen (10 μM). Cells were cultured for 24, 48, and 72 h and cell proliferation was assessed using a sulforhodamine B (SRB) colorimetric assay [18]. The proliferation of cells in the absence of AGE was inhibited by treatment with tamoxifen in both cell lines as expected (Fig. 2e, f). When tamoxifen was added to the cells in the presence of increasing concentration of AGE (from 5 to 50 μg/ml), a dose-dependent restoration of proliferation was observed in both MCF7 and T47D cells (Fig. 2e, f). The data outlined in Fig. 2 identify a mechanistic link between sugar-derived metabolites, AGEs, and ERα regulation which may provide a direct biological consequence of poor lifestyle that may directly impact tamoxifen efficacy.

### Lifestyle intervention reduces the levels of AGE in the circulation of ER+ breast cancer survivors

**Fig. 3:**
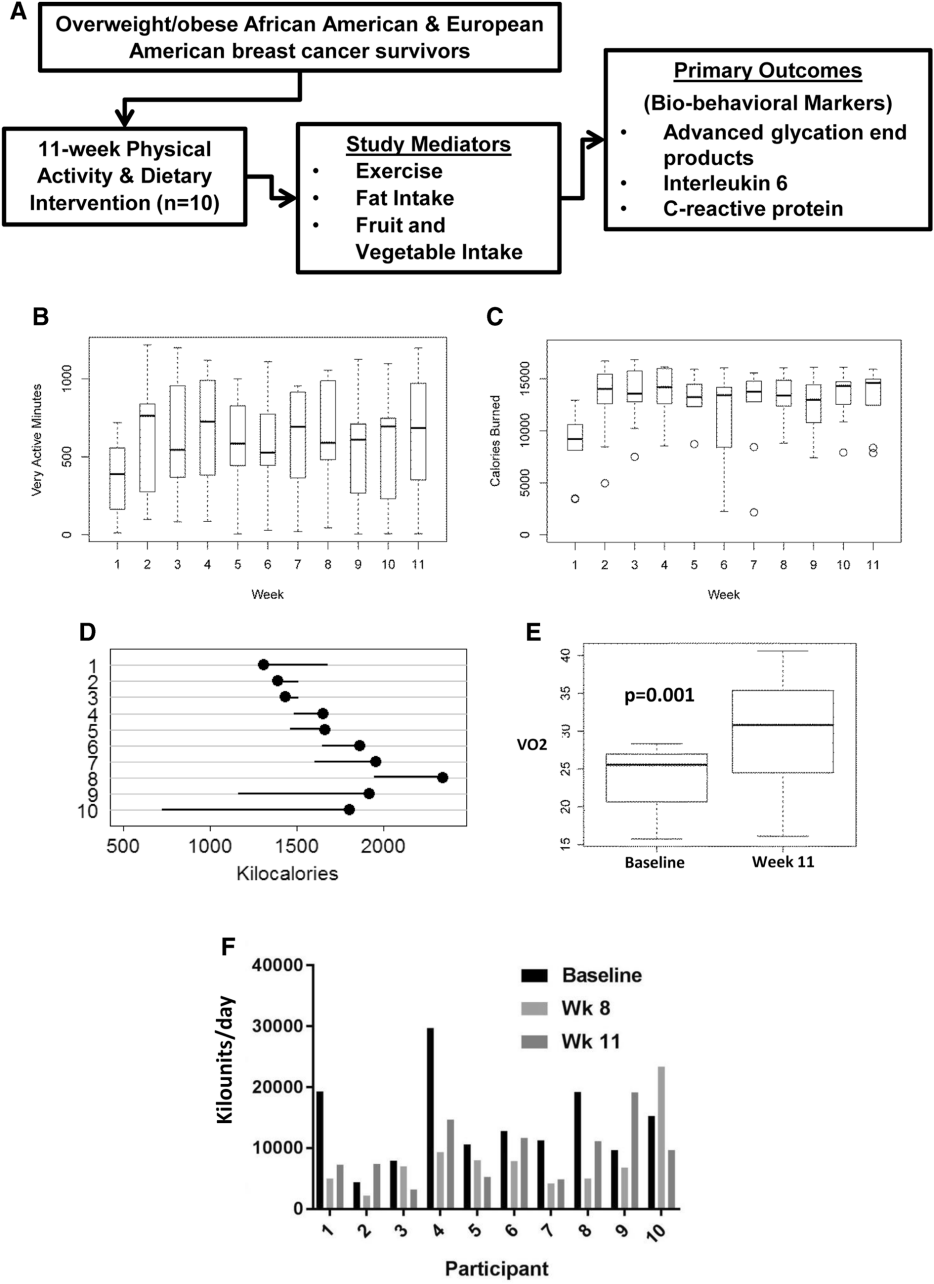
Lifestyle intervention reduces dietary-AGE intake in ER+ breast cancer survivors. **a** Conceptual Framework for the PA and dietary intervention. **b** Average very active minutes achieved by participants during the 11-week lifestyle intervention as assessed from Fitbit data. **c** Average calories burned by participants during the 11-week lifestyle intervention as assessed using 7-day food records. **d** Calorie intake for each participant as assessed using 7-day food records. **e** Average VO2 max at baseline and completion of the 11-week lifestyle intervention. **f** Dietary-AGE intake per participant at baseline, week 8, and week 11 of the lifestyle intervention as assessed using 7-day food records

In a proof of concept pilot study, we tested the ability of a defined lifestyle intervention to lower AGE levels in ten overweight/obese post-menopausal women with non-metastatic stage I–III ER+ breast cancer. Characteristics for the ten participants are summarized in Table 1. The intervention consisted of a focused 11-week physical activity (PA) and dietary counseling intervention that took place within the established clinical setting of the Medical University of South Carolina (MUSC) cardiopulmonary rehabilitation center (Cardiac-Rehab) (Fig. 3a) (Supplementary Methods).

**Table 1:** Participant information for the lifestyle intervention

Characteristic	*n* (%)
Age, mean (range)	56 (46–68) years
Age at diagnosis, mean (range)	57 (46–67) years
Race	
European American	6 (60)
African American	4 (40)
Education level	
< 12 years	1 (10)
High school graduate or GED	1 (10)
Some college	5 (50)
College graduate	1 (10)
Postgraduate	2 (20)
Diabetes	
Yes	1 (10)
No	9 (90)
Time since diagnosis (from date of study registration)	
1–6 months	4 (40)
7–12 months	5 (50)
13–18 months	0 (0)
19–24 months	0 (0)
25–36 months	1 (10)
Tumor stage	
IA	5 (50)
IIA	2 (20)
IIB	2 (20)
IIIA	1 (10)
Menopausal status	
Pre-menopausal	1 (10)
Post-menopausal	9 (90)
Hormone status	
ER/PR+ Her2−	10 (10)
Treatment type all participants had had surgery apart from 1	
Radiation therapy only	1 (10)
Hormone therapy only	1 (10)
Radiation therapy + chemotherapy	1 (10)
Radiation therapy + hormone therapy	4 (40)
Radiation therapy + chemotherapy + hormone therapy	3 (30)

Table of patient characteristics

*GED* general education development, *ER* estrogen receptor, *PR* progesterone receptor, *HER* human EGF receptor

**Fig. 4:**
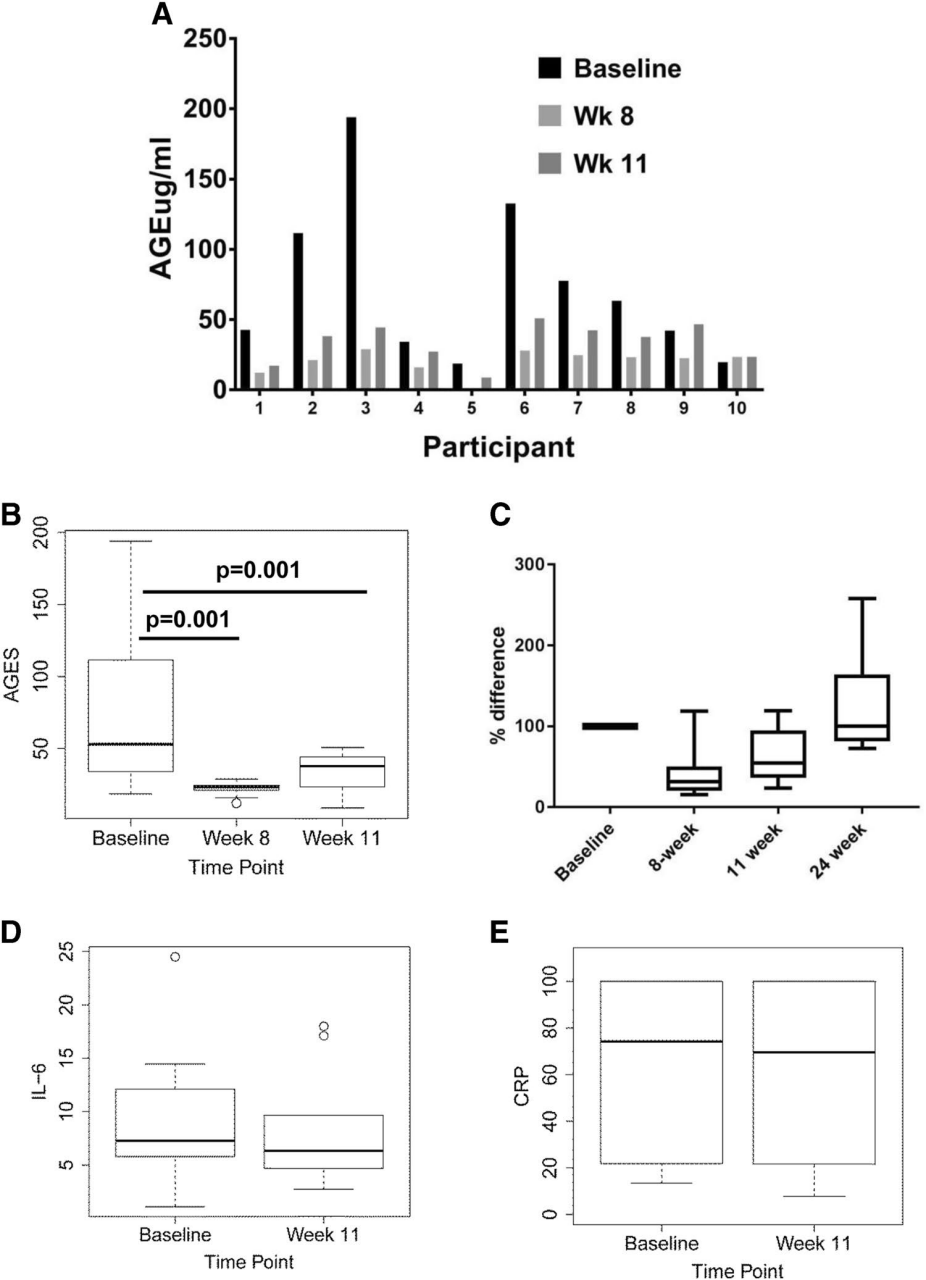
Lifestyle intervention reduces the levels of AGE in the circulation of ER+ breast cancer survivors. **a** Circulatory AGE levels per participant at baseline, week 8 and week 11 of the lifestyle intervention as assessed by ELISA. **b** Average AGE levels at baseline, weeks 8 and 11 for all participants of the lifestyle intervention as assessed by ELISA. **c** Percent change in circulating AGE levels at baseline, weeks 8 and 11 for all participants at of the lifestyle intervention and 13 weeks after the intervention had ended as assessed by ELISA. **d** Average IL6 levels at baseline and at completion for all participants of the 11-week lifestyle intervention as assessed by ELISA. **e** Average CRP levels at baseline and at completion for all participants of the 11-week lifestyle intervention as assessed by ELISA

The pilot study showed a favorable 67% consent rate. Of the 15 enrolled patients, 12 completed the 11-week program; 2 participants were later found to be ineligible because of past weight loss surgery that was not reported at enrollment (and were not included in this analysis). A further 3 dropped out due to competing medical conditions and unwillingness to continue resulting in a 73% adherence rate. Overall adherence to the weekly exercise sessions at cardiac rehab was 82.3% with 50% of participants showing 100% adherence to the twice weekly visits. A modest but general trend of improvement was observed in clinical and laboratory measures as a result of the 11-week intervention (Table 2 and Supplementary Table 1). Statistical significance was observed for diastolic blood pressure (*p* = 0.01) and lipid levels (*p* = 0.05). An analysis of the Fitbit data showed a significant increase in overall very active minutes (Fig. 3b) which coincided with a concurrent increase in average calories burned (Fig. 3c) when compared to baseline assessments, demonstrating the potential health benefits of the study intervention for cancer survivors. Seven (70%) participants showed reduced calorie intake as a result of the dietary intervention while three (30%) increased their calorie intake (Fig. 3d). While some participants showed individual reductions (Supplementary Fig. 2a–c), an analysis of aggregate diet composition showed no significant difference in fat, carbohydrate, and protein composition (Supplementary Fig. 2d). Maximal oxygen consumption (VO2 max) was significantly increased at the end of the intervention when compared to baseline levels (Fig. 3e). An assessment of daily dietary-AGE content was carried out on the basis of 7-day food records; the assessment was estimated from a database of ~ 560 foods and was expressed as AGE Equivalents (Eq/day) (1 AGE equivalent = 1000 kilounits) [21]. Dietary-AGE content was consistently reduced in the participants taking part in the intervention (Fig. 3f). Compared to baseline values, nine participants (90%) showed significant reductions in dietary-AGE intake at week 8, which maintained at week 11 for eight participants (80%). In general, participants that showed significant reductions in dietary-AGE intake at week 8 and week 11 also showed significant reductions in circulating AGE levels when fasting serum samples were analyzed by ELISA (Fig. 4a). Only two patients had higher circulating AGE levels at week 11 than baseline. Median AGE levels at baseline were 53ug/ml compared to 23ug/ml at week 8 and 38 μg/ml at week 11 (Fig. 4b). These findings represent an about 30–60% reduction in the AGE levels. An examination of circulating AGE levels at 13 weeks post intervention (24 weeks) showed a return to pre-intervention levels in all participants (Fig. 4C). Interleukin 6 (IL6) and C-reactive protein (CRP) are often used to assess inflammatory changes induced by diet and exercise. An analysis of IL6 (Fig. 4d) and CRP (Fig. 4e) levels by ELISA in the same AGE-assessed samples revealed no significant differences at any time point.

**Table 2:** Average pre- and post- intervention clinical and laboratory characteristics

	Pre-intervention mean (range)	Post-11-week intervention mean (range)	Paired difference mean	*p* value (paired *t* test)
Clinical characteristics				
Height (cm)	164.3 (160.0–172.7)	164.3 (160.0–172.7)	0.0	
Weight (kg)	90.9 (75.3–110.3)	89.5 (71.5–109.5)	− 1.4	0.34
Pulse (bpm)	80 (67–95)	77 (62–88)	− 2.9	0.31
Respiratory rate (bpm)	17 (16–20)	18 (16–20)	+0.9	0.17
Waist circumference (cm)	107.0 (94.0–124.0)	105.6 (91.4–125.1)	− 1.1	0.50
**H**ip circumference (cm)	116.7 (106.7–125.0)	116.8 (108–141.0)	0.1	0.97
Waist:hip ratio (cm)	0.90 (0.83–0.95)	0.90 (0.83–1.02)	0.0	0.55
Body mass index (kg/m^2^)	33.8 (27.5–43.09)	33.2 (26.4–42.8)	− 0.5	0.30
Systolic blood pressure (mmHg)	137 (117–166)	129 (112–144)	− 7.3	**0.06**
Diastolic blood pressure (mmHg)	84 (74–100)	76 (62–94)	− 8.2	0.01
Laboratory characteristics^a^				
Glucose (mg/dl)^b^	117 (92–139)	117 (102–141)	+ 1.1	0.65
Insulin (mcILI/m1)^c^	24.4 (11.7–43.8)	17.1 (9.1–31.4)	− 4.5	0.09
Lipid Levels (mmol/L)^d^	200 (166–234)	186 (146–236)	− 10.6	**0.049**
Hemoglobin A1C (mmolimo1)^e^	6.1 (5.1–7.6)	6.0 (5.3–7.2)	− 0.1	0.65
HOMA-insulin resistance^f,g^	7.2 (3.5–15.0)	5.1 (2.8–10.2)	− 1.6	**0.11**

The bold numbers are statisticaly significant

Table of clinical and laboratory measures taken during the intervention

^a^24-h fasting blood draws

^b^Glucose: pre-intervention missing = 1 (*n* = 9)

^c^Insulin: pre-intervention missing = 3 (*n* = 7); post-intervention missing = 1 (*n* = 9)

^d^Lipid levels: post-intervention missing = 1 (*n* = 9)

^e^Hemoglobin A1C: post-intervention missing = 1 (*n* = 9)

^f^HOMA-insuling resistance: pre-intervention missing = 4 (*n* = 6); post-intervention missing = 1 (*n* = 9)

^g^HOMA-Insulin resistance formula: ${{\left( {{\text{insulin}} \times {\text{glucose}}} \right)} \mathord{\left/ {\vphantom {{\left( {{\text{insulin}} \times {\text{glucose}}} \right)} {{\text{4}}0{\text{5}}}}} \right. \kern-0pt} {{\text{4}}0{\text{5}}}}$

## Discussion

Given the increasing consumption of dietary-AGEs due to their inherent relationship with a Western lifestyle, it is important to elucidate their potential contribution to the carcinogenic process. Lifestyle and pharmacological interventions that lower AGE levels may then be viable options to reduce breast cancer incidence and improve prognosis in those living with breast cancer. We have identified a mechanistic link between AGEs and ERα regulation which may provide a direct biological consequence of poor lifestyle that may directly impact tamoxifen efficacy.

About 70% of all invasive breast cancers express the estrogen receptor, making it of utmost importance to create more effective treatments, reduce resistance to current treatments, and improve outcomes through lifestyle interventions for these patients. The data presented suggest that AGE levels may be highest in ER+ tumors when compared to ER− and that, at least in vitro, elevated AGE levels can inhibit the growth inhibitory effects of tamoxifen. Studies have shown an association between certain estrogen-independent phosphorylation sites on ERα and tamoxifen resistance [19, 20]. Here, we have shown that two of these residues, ser118 and ser167, are activated in response to AGE exposure and that AGE treatment reduces cell sensitivity to tamoxifen treatment. By identifying an AGE-mediated effect on sensitivity to this drug, we can add to the existing knowledge base that is reinforcing the need for more personalized medicine in cancer, including implementation of specific lifestyle changes. By characterizing a functional role of AGEs in the development of tamoxifen resistance, we suggest that targeted therapies for this type of cancer can potentially become more effective through modification of diet, increased physical activity, and/or through pharmacological strategies to reduce AGE accumulation. While this in vitro work is an important foundation in investigating a role for AGEs in tamoxifen resistance, it fails to address the complexity of the tumor microenvironment and intracellular signaling. However, it is still an important first step and future studies will address the importance of AGE in mediating resistance to tamoxifen especially when those studies use in vivo mouse models to validate the AGE-mediated effects we observed in our cell line studies.

Many of the foods that we consume contribute to the AGE accumulation pool in the body [21–24]. The Western lifestyle consisting of foods high in sugar, protein, and fat and low in fruit, grains, and vegetables is particularly AGE laden and associated with an increased chronic disease risk [21–24]. AGE and AGE precursors are naturally present in uncooked meats; frying, grilling, or roasting leads to a significant increase in AGE formation as we cook our foods [5, 6]. Similarly, food processing and manufacturing also accelerates AGE formation and food manufacturers regularly add AGEs directly during processing in order to improve food appearance and taste 21.

A major goal of the proof of concept lifestyle intervention was to assess the clinical infrastructure of cardiac rehab as a platform for breast cancer survivors. This clinical setting was chosen because the goal of cardiac rehab is to provide supervised exercise training to enable patients with cardiovascular disease to achieve their optimal physical functioning [25–27]. The study shows a favorable 67% consent rate and 73% adherence rate and resulted in an increased level of PA, promoted a healthier diet, and was shown to be a successful strategy for reducing circulating AGE levels in breast cancer patients. This study identifies AGEs as a ubiquitous molecular indicator of lifestyle change. Both dietary and circulating AGE levels were consistently lowered by the cardiac rehab lifestyle intervention, while we observed no change in the more recognized bio-behavioral markers IL6 and CRP.

In summary, AGE metabolite levels may represent a sensitive ubiquitous bio-behavioral marker for the molecular assessment of lifestyle change. Dietary-AGE restriction has been previously shown to reduce circulating AGE levels in patients with chronic kidney disease, metabolic syndrome, and diabetes [28]. As the intervention for this study was a proof of concept exercise, the results should be interpreted with caution due to a number of limitations including the small cohort of participants in the intervention which limits the ability to make conclusions beyond the feasibility of the intervention. However, the present study may provide a defined framework for future studies seeking to improve cancer outcomes and quality of life among cancer survivors across wide racial/ethnic, geographic, and socioeconomic strata.

## Electronic supplementary material

Below is the link to the electronic supplementary material.

Supplementary material 1 (DOCX 482 KB)
