# Supplementary material for: Advanced glycation end products are elevated in estrogen receptor-positive breast cancer patients, alter response to therapy, and can be targeted by lifestyle intervention
Source: Breast Cancer Res Treat. 2018 Oct 27;173(3):559–71. doi: 10.1007/s10549-018-4992-7 (PMC6394600; doi:10.1007/s10549-018-4992-7)
Supplement: Supplementary file 1 — Supplementary material 1 (DOCX 482 KB) [file 10549_2018_4992_MOESM1_ESM.docx]

***SUPPLEMENTARY METHODS***

***Lifestyle Intervention:*** The goal of the PA intervention was for each participant to engage in PA 3-4 days/week, for a total of at least 150 minutes /week (as recommended for cancer survivors by the American Cancer Society and the American College of Sports Medicine). This intervention included 1 weekly supervised and 1 weekly unsupervised session at Cardiac-Rehab [[1](#_ENREF_1)]. The dietary counseling component of the intervention was modeled after the core curriculum of the NIH-initiated Diabetes Prevention Program (DPP) and each participant was provided dietary counseling sessions by a registered dietician [[2](#_ENREF_2)]. Potential participants were identified by clinical coordinators at MUSC-Hollings Cancer Center (HCC) breast cancer clinic through a weekly review of hospital surgery, radiology and pathology reports and clinical referrals to the clinics. Participants consisted of 10 postmenopausal women with non-metastatic Stage I - III BCa who were at least 4 weeks post-treatment and within 24 months of diagnosis. Inclusion and exclusion criteria are documented in supplementary Table 2. The intervention consisted of a focused 11-week physical activity & dietary counseling intervention that will took place within the established clinical setting of Cardiac-Rehab (see Supplementary Methods). The goal of Cardiac-Rehab is to enable participants to achieve their optimal physical, psychological, and social functioning through exercise training [[3](#_ENREF_3)]. Analysis of data from Medicare beneficiaries hospitalized with cardiovascular disease shows a 59% relative risk reduction for mortality for Cardiac-Rehab participants [[3](#_ENREF_3)]. Please see Supplementary methods section for more details.

***Physical activity assessment:*** The goal of the PA Intervention was for each participant to engage in PA 3-4 days/week, for a total of at least 150 minutes (2.5 hours)/week (1 weekly session at Cardiac-Rehab with a staff trainer plus 1 weekly session at Cardiac-Rehab exercising without a trainer) [[1](#_ENREF_1)]. After completing the baseline assessments, each participant received individualized counseling with an exercise physiologist at the Cardiac-Rehab Program and also received a tailored PA prescription, which was based on a combination of Karvonen heart rate (HR) reserve and their Borg rate of perceived exercise (RPE) scores, which were obtained at pretest within the Cardiac-Rehab center [[4](#_ENREF_4),[5](#_ENREF_5)]. Activity was monitored by examining each participant’s Fitbit Zip “Active Minutes” and exercise self-report. The Fitbit Zip measures acceleration and combines user data to calculate steps taken, distance walked, calories burned, and activity duration and intensity [[6](#_ENREF_6),[7](#_ENREF_7)]. Active minutes from Fitbit are based upon activities at or above 3 Metabolic equivalents, active minutes are only awarded after at least ten minutes of continuous moderate to intense PA.

***Dietary assessment:*** The dietary counselling component of the intervention was modeled after the core curriculum of the NIH-initiated DPP Lifestyle Intervention [[2](#_ENREF_2)]. The original DPP lifestyle intervention set goals for participants to achieve and maintain a weight reduction of at least 7 percent of initial body weight through a low-calorie and low-fat diet and to engage in PA of moderate intensity for at least 150 minutes per week. IN the GOAL study, each participant received dietary counseling by a registered dietician along with other key aspects of the DPP such goal setting and self-monitoring to promote healthy eating behaviors that included decreasing fat and calorie intake. Each participant was provided dietary counseling by a registered dietician and stress management education. Key elements of the DC component included clearly defined weight loss goals as well as instruction about nutrition and behavioral self-management.[[8](#_ENREF_8)] Sessions presented information related to modifying energy intake, and how to self-monitor dietary intake. In addition, the dietician obtained data related to BMI (based on height and weight), body fat composition (based on data obtained using Omron body fat monitors), and waist hip ratio (WHR) (using NIH guidelines).

Assessment of daily dietary AGE content was based on 7-day food records, and estimated from a database of ~560 foods which lists AGE values [[9](#_ENREF_9)], and was expressed as AGE Equivalents (Eq/day) (1 AGE equivalent=1000 kilounits). The 7-day food record is based on established guidelines developed to assist in estimating portions [[9](#_ENREF_9)]. This provides an estimate of the amount of food and beverages consumed daily at home or away from home.

***Biomarker analysis:*** Blood was drawn for serum isolation at baseline, 8 and at 11 weeks post intervention. Serum AGE (Cell Biolabs), C-reactive protein (CRP) (R&D Systems) and interleukin 6 (IL6) (R&D Systems) were determined by ELISA at each time point.

**Statistical analysis**

For statistical testing, tissue AGE (two-sided paired Student’s *t*-tests) and circulating AGE (Mann-Whitney test were done using an Excel and GraphPad Prism. Average clinical and laboratory characteristics (Table 2), V02, IL6, and CRP levels were calculated for pre- and 11-weeks post-intervention. One-sample, two-sided t-tests were used to determine if the average paired difference from pre- to 11-weeks post-intervention was different from zero. AGE levels were analyzed using a linear mixed effect regression model with a random component associated with subject ID to account for multiple measurements within subjects over time. Time point was treated categorically, with baseline, week 8, and week 11 as the factor levels. Prior to analysis, AGE values were log-transformed. AGE changes from baseline for weeks 8 and 11, and their 95% CIs, were calculated from the resulting model coefficients and standard errors and exponentiated to calculate the fold-change. Statistical significance was measured at *p* < 0.05.

*REFERENCES*

1. Prevention CfDCa Physical Activity. <http://www.cdc.gov/physicalactivity/everyone/guidelines/adults.html>. Accessed September 24 2014

2. Group TDPPR (2002) The diabetes prevention program (DPP): description of lifestyle intervention. Diabetes care 25 (12):2165-2171

3. Suaya JA, Stason WB, Ades PA, Normand SL, Shepard DS (2009) Cardiac rehabilitation and survival in older coronary patients. Journal of the American College of Cardiology 54 (1):25-33. doi:10.1016/j.jacc.2009.01.078

4. Braun LT WN, Rosenson RS Components of cardiac rehabilitation and exercise prescription. Wolters Kluwer Health. <http://www.uptodate.com/contents/components-of-cardiac-rehabilitation-and-exercise-prescription>. Accessed September 24 2014

5. Scherr J WB, Christle W, Pressler A, Wagenpfeil S, Halle M (2012) Associations between Borg's rating of perceived exertion and physiological measures of exercise intensity. Eur J Appl Physiol:1-9

6. Sasaki JE, Hickey A, Mavilia M, Tedesco J, John D, Kozey Keadle S, Freedson PS (2014) Validation of the Fitbit Wireless Activity Tracker(R) for Prediction of Energy Expenditure. Journal of physical activity & health. doi:10.1123/jpah.2012-0495

7. Takacs J, Pollock CL, Guenther JR, Bahar M, Napier C, Hunt MA (2014) Validation of the Fitbit One activity monitor device during treadmill walking. Journal of science and medicine in sport / Sports Medicine Australia 17 (5):496-500. doi:10.1016/j.jsams.2013.10.241

8. Knowler WC, Barrett-Connor E, Fowler SE, Hamman RF, Lachin JM, Walker EA, Nathan DM (2002) Reduction in the incidence of type 2 diabetes with lifestyle intervention or metformin. N Engl J Med 346 (6):393-403. doi:10.1056/NEJMoa012512

9. Uribarri J, Woodruff S, Goodman S, Cai W, Chen X, Pyzik R, Yong A, Striker GE, Vlassara H (2010) Advanced glycation end products in foods and a practical guide to their reduction in the diet. J Am Diet Assoc 110 (6):911-916 e912. doi:S0002-8223(10)00238-5 [pii]

10.1016/j.jada.2010.03.018

**Supplementary Figure Legends**

**Supplementary Figure 1. A.** Western blot analysis of time dependent AKT and ERK phosphorylation after treatment with AGE (50ug/mL) in T47D breast cancer cells. **B.** Western blot analysis of ERα phosphorylation after treatment with AGE (50ug/mL) in T47D breast cancer cells. **C.** Western blot analysis of time dependent ERα phosphorylation after treatment with AGE (50ug/mL) in MCF7 cells. **D.** Western blot analysis of time dependent ERα phosphorylation after treatment with AGE (50ug/mL) in T47D cells. **E.** Western blot analysis of AKT and ERK phosphorylation after treatment with AGE (50ug/mL) in T47D cells in the presence of AKT inhibitor. **F.** Western blot analysis of AKT and ERK phosphorylation after treatment with AGE (50ug/mL) in T47D cells in the presence of ERK inhibitor.

**Supplementary Table 1.** Pre- and post- intervention weight and BMI changes for each participant.

**Supplementary Table 2.** Inclusion and exclusion criteria for the lifestyle intervention.

**Supplementary Figure 2. A.** Protein intake at baseline and 11 weeks for each individual participant as assessed using 7 day food records. **B.** Carbohydrate intake at baseline 11 weeks for each individual participant as assessed using 7 day food records. **C.** Fat intake at baseline 11 weeks for each individual participant as assessed using 7 day food records. **D.** Average protein, carbohydrate and fat intake at baseline, week 8 and week 11 of the lifestyle intervention as assessed using 7 day food records.

***

***

**Supplementary Table 1**


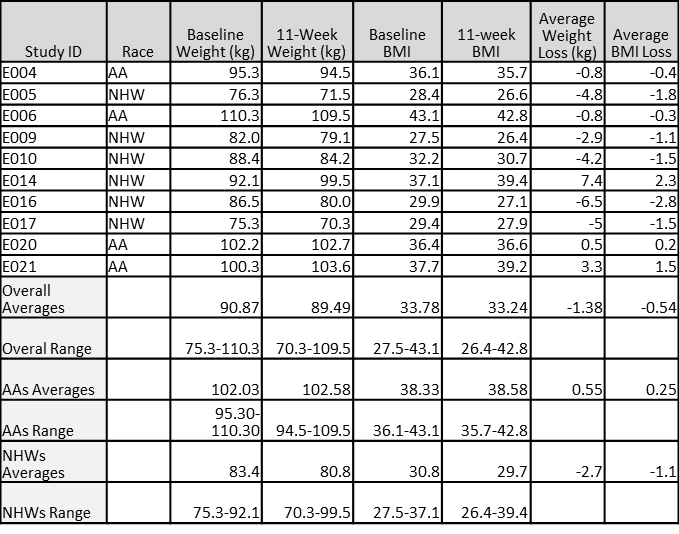


**Supplementary Table 2**

INCLUSION CRITERIA

1. Subjects must have had histologically confirmed invasive breast cancer with TNM stage pT1-3, pN0-3, M0. Bilateral breast cancer is allowed.

2. Subjects must be enrolled within 36 months of the first histologic diagnosis. (e.g. date of the initial core biopsy date)

3. Subjects must have had definitive surgical treatment for invasive disease.

4. Subjects may have received standard adjuvant loco-regional radiation, adjuvant endocrine treatment, trastzumab or other biologics or bisphosphonates prior to or during study intervention.

5. Subjects may have received chemotherapy or radiation therapy 4 weeks or more prior to study entry.

6. Known estrogen and progesterone receptor status and Her-2 status.

7. BMI > 25.

8. ECOG Performance status 0, 1 or 2.

9. Participants must be age > 18 and < 75 and have a life expectancy of at least 5 years.

10. Participants must be accessible for treatment and follow-up and must sign informed consent.

11. Subjects with known diabetes are permitted. However, they must be under active treatment for this condition.

12. Prior physical therapy assessment for instruction on post breast surgery range of motion exercises is allowed.

EXCLUSION CRITERIA

1. Subjects who are pregnant or plan pregnancy are not eligible.

2. Subjects who plan enrollment in other weight loss or exercise programs during the 12 weeks of study participation.

3. Subjects must not have received chemotherapy within 4 weeks of enrollment and may not undergo chemotherapy during the study intervention.

4. Patients who have had previous weight loss surgery (specifically gastric bypass, sleeve gastrectomy and biliary pancreatic diversion) are excluded.

5. Patients who have previously participated in cardiopulmonary rehabilitation are excluded.

6. Patients who have constrained mobility secondary to problems with balance, bone and/or joint disease are not eligible.

7. Patients who have unstable angina or cardiovascular disease which prohibits them from exercise are ineligible.





***SUPPLEMENTARY METHODS***

***Lifestyle Intervention:*** The goal of the PA intervention was for each participant to engage in PA 3-4 days/week, for a total of at least 150 minutes /week (as recommended for cancer survivors by the American Cancer Society and the American College of Sports Medicine). This intervention included 1 weekly supervised and 1 weekly unsupervised session at Cardiac-Rehab [[1](#_ENREF_1)]. The dietary counseling component of the intervention was modeled after the core curriculum of the NIH-initiated Diabetes Prevention Program (DPP) and each participant was provided dietary counseling sessions by a registered dietician [[2](#_ENREF_2)]. Potential participants were identified by clinical coordinators at MUSC-Hollings Cancer Center (HCC) breast cancer clinic through a weekly review of hospital surgery, radiology and pathology reports and clinical referrals to the clinics. Participants consisted of 10 postmenopausal women with non-metastatic Stage I - III BCa who were at least 4 weeks post-treatment and within 24 months of diagnosis. Inclusion and exclusion criteria are documented in supplementary Table 2. The intervention consisted of a focused 11-week physical activity & dietary counseling intervention that will took place within the established clinical setting of Cardiac-Rehab (see Supplementary Methods). The goal of Cardiac-Rehab is to enable participants to achieve their optimal physical, psychological, and social functioning through exercise training [[3](#_ENREF_3)]. Analysis of data from Medicare beneficiaries hospitalized with cardiovascular disease shows a 59% relative risk reduction for mortality for Cardiac-Rehab participants [[3](#_ENREF_3)]. Please see Supplementary methods section for more details.

***Physical activity assessment:*** The goal of the PA Intervention was for each participant to engage in PA 3-4 days/week, for a total of at least 150 minutes (2.5 hours)/week (1 weekly session at Cardiac-Rehab with a staff trainer plus 1 weekly session at Cardiac-Rehab exercising without a trainer) [[1](#_ENREF_1)]. After completing the baseline assessments, each participant received individualized counseling with an exercise physiologist at the Cardiac-Rehab Program and also received a tailored PA prescription, which was based on a combination of Karvonen heart rate (HR) reserve and their Borg rate of perceived exercise (RPE) scores, which were obtained at pretest within the Cardiac-Rehab center [[4](#_ENREF_4),[5](#_ENREF_5)]. Activity will be monitored by examining each participant’s Fitbit Zip “Active Minutes” and exercise self-report. The Fitbit Zip measures acceleration and combines user data to calculate steps taken, distance walked, calories burned, and activity duration and intensity [[6](#_ENREF_6),[7](#_ENREF_7)]. Active minutes from Fitbit are based upon activities at or above 3 Metabolic equivalents, active minutes are only awarded after at least ten minutes of continuous moderate to intense PA.

***Dietary assessment:*** The dietary counselling component of the intervention was modeled after the core curriculum of the NIH-initiated DPP Lifestyle Intervention [[2](#_ENREF_2)]. Each participant was provided dietary counseling by a registered dietician and stress management education. Key elements of the DC component included clearly defined weight loss goals as well as instruction about nutrition and behavioral self-management.[[8](#_ENREF_8)] Sessions presented information related to modifying energy intake, and how to self-monitor dietary intake. In addition, the dietician obtained data related to BMI (based on height and weight), body fat composition (based on data obtained using Omron body fat monitors), and waist hip ratio (WHR) (using NIH guidelines).

Assessment of daily dietary AGE content was based on 7-day food records, and estimated from a database of ~560 foods which lists AGE values [[9](#_ENREF_9)], and was expressed as AGE Equivalents (Eq/day) (1 AGE equivalent=1000 kilounits). The 7-day food record is based on established guidelines developed to assist in estimating portions [[9](#_ENREF_9)]. This provides an estimate of the amount of food and beverages consumed daily at home or away from home.

***Biomarker analysis:*** Blood was drawn for serum isolation at baseline, 8 and at 11 weeks post intervention. Serum AGE (Cell Biolabs), C-reactive protein (CRP) (R&D Systems) and interleukin 6 (IL6) (R&D Systems) were determined by ELISA at each time point.

**Statistical analysis**

For statistical testing, tissue AGE (two-sided paired Student’s *t*-tests) and circulatory AGE (Mann-Whitney test were done using an Excel and GraphPad Prism. Average clinical and laboratory characteristics (Table 2), V02, IL6, and CRP levels were calculated for pre- and 11-weeks post-intervention. One-sample, two-sided t-tests were used to determine if the average paired difference from pre- to 11-weeks post-intervention was different from zero. AGE levels were analyzed using a linear mixed effect regression model with a random component associated with subject ID to account for multiple measurements within subjects over time. Time point was treated categorically, with baseline, week 8, and week 11 as the factor levels. Prior to analysis, AGE values were log-transformed. AGE changes from baseline for weeks 8 and 11, and their 95% CIs, were calculated from the resulting model coefficients and standard errors and exponentiated to calculate the fold-change. Statistical significance was measured at *p* < 0.05.

*REFERENCES*

1. Prevention CfDCa Physical Activity. <http://www.cdc.gov/physicalactivity/everyone/guidelines/adults.html>. Accessed September 24 2014

2. Group TDPPR (2002) The diabetes prevention program (DPP): description of lifestyle intervention. Diabetes care 25 (12):2165-2171

3. Suaya JA, Stason WB, Ades PA, Normand SL, Shepard DS (2009) Cardiac rehabilitation and survival in older coronary patients. Journal of the American College of Cardiology 54 (1):25-33. doi:10.1016/j.jacc.2009.01.078

4. Braun LT WN, Rosenson RS Components of cardiac rehabilitation and exercise prescription. Wolters Kluwer Health. <http://www.uptodate.com/contents/components-of-cardiac-rehabilitation-and-exercise-prescription>. Accessed September 24 2014

5. Scherr J WB, Christle W, Pressler A, Wagenpfeil S, Halle M (2012) Associations between Borg's rating of perceived exertion and physiological measures of exercise intensity. Eur J Appl Physiol:1-9

6. Sasaki JE, Hickey A, Mavilia M, Tedesco J, John D, Kozey Keadle S, Freedson PS (2014) Validation of the Fitbit Wireless Activity Tracker(R) for Prediction of Energy Expenditure. Journal of physical activity & health. doi:10.1123/jpah.2012-0495

7. Takacs J, Pollock CL, Guenther JR, Bahar M, Napier C, Hunt MA (2014) Validation of the Fitbit One activity monitor device during treadmill walking. Journal of science and medicine in sport / Sports Medicine Australia 17 (5):496-500. doi:10.1016/j.jsams.2013.10.241

8. Knowler WC, Barrett-Connor E, Fowler SE, Hamman RF, Lachin JM, Walker EA, Nathan DM (2002) Reduction in the incidence of type 2 diabetes with lifestyle intervention or metformin. N Engl J Med 346 (6):393-403. doi:10.1056/NEJMoa012512

9. Uribarri J, Woodruff S, Goodman S, Cai W, Chen X, Pyzik R, Yong A, Striker GE, Vlassara H (2010) Advanced glycation end products in foods and a practical guide to their reduction in the diet. J Am Diet Assoc 110 (6):911-916 e912. doi:S0002-8223(10)00238-5 [pii]

10.1016/j.jada.2010.03.018
